# Spanish Word Meaning in Mobile Toolbox (MTB): Association with Language Preferences in Different Contexts

**DOI:** 10.1093/geroni/igaf122.3236

**Published:** 2025-12-31

**Authors:** Jiwon Kim, Elizabeth Dworak, Y Catherine Han, Miriam Novack, Cindy Nowinski, Zahra Hosseinian, Richard Gershon

**Affiliations:** Northwestern University, Evanston, Illinois, United States; Northwestern University, Evanston, Illinois, United States; Northwestern University, Evanston, Illinois, United States; Northwestern University, Evanston, Illinois, United States; Northwestern University, Evanston, Illinois, United States; Northwestern University, Evanston, Illinois, United States; Northwestern University, Evanston, Illinois, United States

## Abstract

Given that Spanish is the second most spoken language in the US, the lack of cognitive assessments in Spanish for older adults presents a significant gap in clinical care and research for both Spanish-speakers and Spanish-English bilinguals. For bilinguals in particular, the optimal assessment language remains unclear, particularly as their language use and preferences may vary across contexts. The Mobile Toolbox (MTB), available in English and Spanish, provides brief, sensitive, cognitive measures for assessing functioning across the lifespan. In this study, we examined the association between language preference in various contexts and vocabulary scores on the MTB Spanish Word Meaning using regression models with orthogonal contrast codes. The sample included 1,620 Spanish-speakers aged 18-64 (M = 42.79, SD = 14.45), all of whom chose to complete all assessment materials in Spanish. Results revealed that preferring to speak English at home was negatively associated with Spanish vocabulary performance (beta=-.27). Conversely, English preferences in work communication (beta=.37), reading official documents (beta=0.35), media consumption (e.g., TV: beta=.59, news: beta=.36), and smartphone settings (beta=.21) were positively associated with Spanish vocabulary scores. English preferences for reading personal content (books) and community communication did not predict vocabulary performance. In sum, these findings suggest language engagement between home and work settings differentially affect Spanish vocabulary performance in Spanish bilinguals. Future work may examine this effect across different MTB assessments (e.g., executive functioning) as well as in clinical samples with dementia-related diagnoses.

